# Intramuscular myxoma of the supinator muscle causing posterior interosseous nerve palsy: a case report and review of literature

**DOI:** 10.1093/jscr/rjaf1017

**Published:** 2025-12-22

**Authors:** Motaz AlAqeel, Mishari Alanezi, Nouf Alabdulkarim, Fahad Almehrij, Norah Alromaih

**Affiliations:** Department of Orthopedic Surgery, College of Medicine, King Saud University, PO Box 2925, Riyadh 11461, Saudi Arabia; College of Medicine, King Saud University, PO Box 2925, Riyadh 11461, Saudi Arabia; Department of Orthopedic Surgery, College of Medicine, King Saud University, PO Box 2925, Riyadh 11461, Saudi Arabia; Orthopedic Department, Operation Program of Medical Cities, Ministry of Interior, PO Box 2925, Riyadh 11461, Saudi Arabia; Orthopaedic Surgery Resident, King Saud Medical City, PO Box 2925, Riyadh 11461, Saudi Arabia

**Keywords:** intramuscular myxoma, posterior interosseous nerve palsy, wrist drop

## Abstract

Intramuscular myxomas (IMMs) are rare benign tumors of large skeletal muscles, most often in the thigh, shoulder, or gluteal region. Forearm involvement, especially in the supinator, is exceptionally uncommon and may cause nerve compression when near neurovascular structures. We report a rare case of a 47-year-old woman presenting with a gradually enlarging, painless mass in the dorsal aspect of the proximal left forearm and associated wrist drop. Imaging revealed a well-circumscribed, mucinous lesion within the supinator muscle. Histopathological evaluation confirmed the diagnosis of an IMM. The tumor was surgically excised, with intraoperative findings revealing compression of the posterior interosseous nerve. Postoperatively, the patient demonstrated progressive improvement in wrist extension without evidence of recurrence. This case highlights a rare presentation of IMM causing posterior interosseous nerve compression and wrist drop. Marginal excision is often curative given its benign nature. IMM should be considered in painless, slow-growing masses with motor deficits.

## Introduction

Myxomas are benign mesenchymal tumors characterized by gelatinous myxoid stroma and hypocellularity [1]. While they are most commonly encountered in the heart, constituting over 85% of all benign primary cardiac tumors [2], they can also arise in extracardiac soft tissues, where they can exhibit distinct clinical and pathological features. Among the extracardiac variants, intramuscular myxomas (IMMs) are an extremely rare subset, with an incidence rate of 0.13 per 100 000 [3]. IMM commonly originates within the large skeletal muscles and is typically found in the thigh, gluteal region, or shoulder girdle, with very limited cases reported in the forearm [4].

Clinically, IMMs often present as slow-growing, painless masses that are frequently discovered incidentally [5]. Although they are typically asymptomatic, IMM can occasionally produce symptoms due to mass effect; in rare instances, when presenting in confined anatomical compartments or adjacent to neurovascular structures, they can cause nerve compression syndrome [6]. In extremely rare cases where IMM arises within or adjacent to the supinator muscle of the proximal forearm, posterior interosseous nerve (PIN) palsy can occur, resulting in wrist or finger extension weakness, and in more advanced cases, complete wrist drop can occur [7]. Early diagnosis and timely surgical intervention are critical to prevent permanent functional deficits. Histologically, IMMs are defined by their hypocellular and hypovascular nature, composed of bland spindle or stellate cells embedded in a myxoid matrix [8].

In this article, we present a rare case of an IMM arising from the supinator muscle in a middle-aged woman. The patient provided informed consent to publish her medical information for academic and educational purposes.

## Case report

A 47-year-old right-handed female with a medical history of hypothyroidism presented to the outpatient clinic with a gradually enlarging, painless swelling over the dorsal aspect of the proximal left forearm, which she had first noticed approximately 9 months prior to presentation. The swelling had developed insidiously and increased following upper limb exercise. She denied any history of trauma, fever, weight loss, night sweats, or other constitutional symptoms.

On physical examination, a firm, non-tender, and mobile mass was palpable over the posterolateral aspect of the proximal forearm. The overlying skin was freely mobile, and there were no signs of local inflammation. Neurological examination revealed difficulty with active wrist extension, consistent with weakness in the distribution of the PIN. However, there were no sensory deficits, and the distal neurovascular status remained intact.

**Figure 1:**
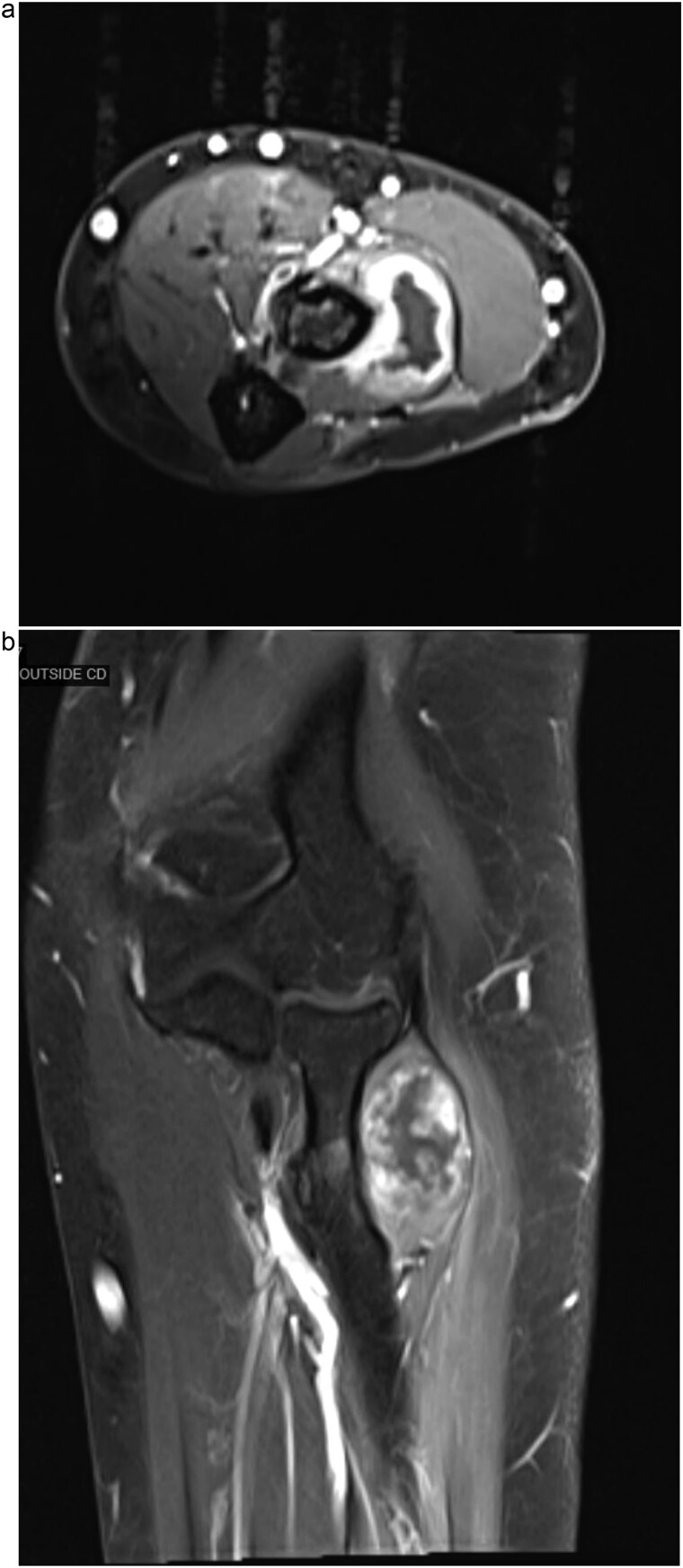
(a) Axial MRI of the forearm demonstrating a mass within the supinator muscle, associated with compression of the PIN. (b) Coronal MRI of the forearm showing a well-defined intramuscular mass within the supinator muscle, with surrounding edema and displacement of adjacent neurovascular structures.

The clinical findings raised concern for a space-occupying lesion causing nerve compression, and further diagnostic workup was initiated with magnetic resonance imaging (MRI), which revealed a well-defined, oval-shaped intramuscular mass located within the supinator muscle, measuring 3.2 × 2.1 × 1.8 cm. The lesion demonstrated homogeneously low-to-intermediate signal intensity on T1-weighted images and markedly high signal intensity on T2-weighted images, consistent with its high mucinous content. Post-contrast images showed mild to moderate peripheral or septal enhancement, with no evidence of infiltration into adjacent structures (Fig. 1).

A percutaneous core needle biopsy was subsequently performed under general anesthesia. Histopathological examination confirmed the diagnosis of a low-grade intramuscular myxoid neoplasm, consistent with an IMM. Subsequently, the patient underwent excision of the left forearm myxoid tumor under regional anesthesia. A tourniquet was applied, and the operative limb was prepped and draped in a sterile fashion. Using Kaplan’s approach, a skin incision was made, and dissection was carried out layer by layer to expose the extensor muscles.

Intraoperatively, the mass was located beneath the supinator muscle, deep to the extensor carpi radialis brevis (ECRB), and was found to be compressing only the PIN distal to the ECRB branch, while the sensory branch of the radial nerve remained intact. The compressed segment of the PIN appeared flattened but maintained continuity and normal caliber distally following decompression. The tumor was situated within the deep fibers of the supinator muscle, which were split but not infiltrated. The fibers were gently separated, and the mass was excised en bloc without sacrificing the muscle. The wound was irrigated with hydrogen peroxide and povidone-iodine, and hemostasis was achieved. During her routine follow-up visits at 3, 6, and 9 months, she demonstrated substantial improvement in wrist extension, with no clinical or radiological evidence of tumor recurrence.

## Discussion

IMMs predominantly occur between 40 and 70 years old, with a notable female predominance [9]. The clinical presentation is often subtle and nonspecific. Typically, patients with IMMs present with a slowly enlarging, painless soft tissue mass, frequently discovered incidentally [10]. Over half of the cases remain asymptomatic until the tumor reaches a considerable size. When symptoms do occur, they are usually related to compression of nearby structures, resulting in pain, paresthesia, or motor weakness [11].

Nerve compression is a rare but clinically significant manifestation of IMMs. When the tumor arises in confined anatomical spaces, such as the forearm, pelvic cavity, or deep compartments of the thigh, it can compress adjacent nerves, leading to neuropathic symptoms [6]. In particular, when located in the proximal forearm, IMMs can involve the supinator muscle, resulting in PIN compression. This may manifest as motor weakness of finger and wrist extensors, and in more advanced cases, it can lead to complete wrist drop [7]. Therefore, early diagnosis and management are crucial to prevent permanent functional deficits in patients presenting with neurologic signs.

The diagnosis of IMM requires a high index of clinical suspicion, as it can closely mimic a variety of benign soft tissue lesions. MRI is essential in the evaluation and plays an important role in both diagnosis and surgical planning [12]. IMMs typically demonstrate well-circumscribed margins with no evidence of local invasion, distinguishing them from more aggressive lesions [13]. The classic MRI features include homogeneously low-to-intermediate signal intensity on T1-weighted sequences and markedly high signal intensity on T2-weighted images, reflecting the tumor’s high mucinous content [14]. These characteristic radiological findings, along with the absence of surrounding tissue invasion, are highly suggestive of a benign lesion. Histologically, IMMs are characterized by hypocellular, hypovascular lesions composed of bland spindle or stellate cells embedded in a gelatinous myxoid matrix [12]. The cells typically have small, uniform nuclei, with no cytologic atypia, mitotic activity, or necrosis, distinguishing them from low-grade sarcomas.

Management of IMMs is primarily surgical. Complete surgical excision with clear margins is the treatment of choice and is usually curative. Marginal excision is often sufficient due to the tumor’s benign nature and well-circumscribed morphology. In a large series, no local recurrences were reported following either marginal or wide excision, supporting conservative resection when the diagnosis is confirmed [5]. Similarly, a study by Baltu *et al.* [15] reported no recurrence in nine patients with IMMs at various anatomical sites treated with simple excision during a mean follow-up of 39 months, further validating the safety and effectiveness of limited surgical approaches. Wide excision may be reserved for cases where the diagnosis is uncertain or malignancy cannot be excluded preoperatively [5].

## Conclusion

This rare forearm IMM caused PIN compression and wrist drop, highlighting the importance of considering soft tissue masses with neurological deficits and the value of early diagnosis and surgical excision for functional recovery.

## Data Availability

This study did not create new data; therefore, data sharing is not applicable.
